# Microporous Implants Modified by Bifunctional Hydrogel with Antibacterial and Osteogenic Properties Promote Bone Integration in Infected Bone Defects

**DOI:** 10.3390/jfb14040226

**Published:** 2023-04-16

**Authors:** Yiping Pu, Xuecai Lin, Qiang Zhi, Shichong Qiao, Chuangqi Yu

**Affiliations:** 1 Department of Oral Surgery, Shanghai Ninth People’s Hospital, College of Stomatology, Shanghai Jiao Tong University School of Medicine, Shanghai 200001, China; 2 National Clinical Research Center for Oral Diseases, Shanghai 200011, China; 3 Shanghai Key Laboratory of Stomatology & Shanghai Research Institute of Stomatology, Shanghai 200001, China; 4 Research Unit of Oral and Maxillofacial Regenerative Medicine, Chinese Academy of Medical Sciences, Shanghai 200001, China; 5 Hongqiao Community Health Service Center, Minhang District, Shanghai 201103, China; 6 Department of Implant Dentistry, Shanghai Ninth People’s Hospital, College of Stomatology, Shanghai Jiao Tong University School of Medicine, Shanghai Jiao Tong University, Shanghai 200240, China; 7 Shanghai Key Laboratory of Stomatology, National Center for Stomatology, Shanghai 200011, China

**Keywords:** bacterial infection, implant, hydrogel, antibacterial, bone integration

## Abstract

Prosthesis implantation and bone integration under bacterial infection are arduous challenges in clinical practice. It is well known that the reactive oxygen species (ROS) produced by bacterial infection around the bone defects will further hinder bone healing. To solve this problem, we prepared a ROS-scavenging hydrogel by cross-linking polyvinyl alcohol and a ROS-responsive linker, N^1^-(4-boronobenzyl)-N^3^-(4-boronophenyl)-N^1^, N^1^, N^3^, N^3^-tetramethylpropane-1, 3-diaminium, to modify the microporous titanium alloy implant. The prepared hydrogel was used as an advanced ROS-scavenging tool to promote bone healing by inhibiting the ROS levels around the implant. Bifunctional hydrogel serving as a drug delivery system can release therapeutic molecules, including vancomycin, to kill bacteria and bone morphogenetic protein-2 to induce bone regeneration and integration. This multifunctional implant system that combines mechanical support and disease microenvironment targeting provides a novel strategy for bone regeneration and integration of implants in infected bone defects.

## 1. Introduction

Promoting infected bone healing and peri-implant bone integration in infected bone defects is a challenge in clinical practice, and there are still no ideal and potent bone substitutes to solve this orthopedic problem perfectly [1]. Injured regions and artificial implants are beneficial for bacterial adhesion and subsequent biofilm formation, thereby accelerating the infected bone progress [2,3]. Although considerable progress has been made in the development of various bone implants over the past decades, peri-prosthetic infection caused by bacteria is a major factor leading to implant failure [4]. Under physiological conditions, most reactive oxygen species (ROS), which primarily include hydrogen peroxide (H_2_O_2_), superoxide anions (O_2_^−^), and hydroxyl radicals (HO^•^) are produced through the intracellular mitochondrial respiratory chain pathway. Antioxidants produced by the body can effectively neutralize ROS or repair ROS-induced tissue damage, thereby offsetting the harmful effects of oxidative stress. When infection and inflammation occur, ROS production increases sharply, high levels or high activity of ROS cannot be balanced by the antioxidant defense system, and excess ROS leads to oxidative stress and tissue damage. Under this infected microenvironment, the uncontrolled accumulation ROS can induce oxidative stress and inhibit viability of endogenous cells, and inactivate growth factors in the damaged tissue, thereby profoundly compromising their regenerative potential [5,6]. Titanium and its alloys, the most commonly used materials in implants without intrinsic antibacterial properties, are susceptible to bacterial invasion and colonization, thus leading to severe clinical complications such as peri-prosthetic inflammation, implant loosening and detachment, and osteomyelitis [7,8]. Once bacterial infection emerges around the prosthesis, bone regeneration and integration are inhibited by processive infection and inflammatory response, leading to the failure of implant transplantation. Thus, alternative bioactive materials with dual-functional properties of antibacterial and osteogenic induction-modified titanium alloy implants are novel therapeutic strategies for peri-implant bone integration in infected bone defects.

Hydrogels are generally cross-linked by hydrophilic polymer chains in aqueous microenvironments and have wide application prospects as drug carriers and tissue engineering substrates [9,10]. They are considered alternative materials for preparing tissue engineering scaffolds owing to their desirable instinctive attributes, including simplified structure, physiological-mimic circumstance, animated mechanical potency, and optimized nutrition permeability [11]. Biomimetic engineering hydrogels with three-dimensional (3D) networks possess various physical characteristics homologous to the native extracellular matrix (ECM), such as water retention, biocompatibility, excellent permeability, plasticity, elasticity, and low interfacial tension [12,13]. In addition to serving as an artificial ECM, hydrogels are widely used as drug delivery systems. Hydrogels prepared from natural or synthetic polymers have been found to extend the retention time of encapsulated drugs and achieve sustained-release profiles. The therapeutic effects of bioactive hydrogels in tissue repair mainly depend on their physicochemical characteristics and drug-release profiles [14,15]. As a drug delivery system, hydrogels can extend the retention time of encapsulated drugs for sustainable release to obtain a better therapeutic effect than systemic administration [16]. These advantages indicate that hydrogels have the potential to load multifunctional ingredients in the treatment of infected bone defects. In recent years, researchers not only focus on simple anti-infection, but also desire to design hydrogels with dual-function of antibacterial and osteogenesis to achieve the optimal effect for reconstruction of bone defects in infection. Biomaterials with inherent osteogenic induction ability have been applied for infected bone defects [17,18]. The composite of mineralization components and hydrogels loaded with antibacterial agents inhibit bacterial infection and induce bone regeneration in infected bone defects [19,20]. Considering the required sufficient mechanical support in load-bearing sites during repair, another hydrogel formulation incorporating silver nanoparticles and silica was filled into 3D-printed titanium microporous implants with good mechanical properties. The composite implants provided sufficient mechanical strength while inhibiting bacterial proliferation and promoting bone integration at the prosthesis interface in periprosthetic infection [21]. However, in the infected bone defect, the excessive ROS from bacterial infection accumulated in the tissue can not only lead to strong inflammatory responses to cause bone vulnerable, but also impair the functions of endogenous stem cells and macrophages for tissue repair [22]. Therefore, in addition to loading osteogenic and antibacterial drugs, hydrogels with active oxygen scavenging ability have attracted researchers’ interest.

In this study, we prepared a hydrogel as a drug-delivery system by cross-linking polyvinyl alcohol (PVA) with N1-(4-boronobenzyl)-N3-(4-boronophenyl)-N1, N1, N3, N3-tetramethylpropane-1, 3-diaminium (TPA). PVA is a biodegradable, water-soluble polymer with a large number of hydroxyl groups in its molecular chain, so it has good water solubility, film forming, adhesion, and thermal stability. At the same time, because of its good mechanical properties, chemical stability, and good compatibility with human tissues, it is widely used to prepare bone tissue engineering materials [23,24]. The ROS-responsive linker, TPA, which can scavenge ROS, indicated an effect in promoting tissue repair in an infectious microenvironment [22]. This characteristic of TPA provides a possibility to improve the microenvironment of ROS accumulation in infected bone defects, thus paving the way for the drugs released from hydrogels to play a full role. Vancomycin, the classic antibiotic for an orthopedic infection, and bone morphogenetic protein-2 (BMP-2), an osteogenic inducible factor, were encapsulated into the hydrogel to realize dual functions. The drug-loaded hydrogel was used as a modifier to fill 3D-printed microporous titanium alloy implants, construct a composite system, and transplant into infected bone defects. This study aimed to investigate the dual functions of this organic-inorganic composite implant in inhibiting bacteria and promoting osteogenesis in vitro and in vivo in an infected microenvironment. This multifunctional implant system that combines mechanical support and disease microenvironment targeting provides a novel strategy for bone regeneration and integration of implants in infected bone defects.

## 2. Materials and Methods

### 2.1. Materials

Vancomycin, PVA, N, N, N′, N′-tetramethyl-1,3-propanediamine, and 4-(bromomethyl) phenylboronic acid were supplied by Macklin. BMP-2 was supplied by R&D Systems. Hydrogen peroxide (H_2_O_2_, 30 wt%) was supplied by Beijing Chemical Works (Beijing, China). The strains of *Staphylococcus aureus* (*S. aureus,* ATCC 29213) and methicillin-resistant *S. aureus* (*MRSA,* ATCC 33591) were obtained from the American Type Culture Collection (ATCC, Manassas, VA, USA). Rabbit bone marrow mesenchymal stem cells (BMSCs), osteogenic differentiation medium for rabbit BMSCs, and Alizarin Red stain were supplied by Procell (Wuhan, China). Low-glucose Dulbecco’s Modified Eagle’s medium (LG-DMEM) and streptomycin-penicillin were supplied by Gibco^®^ Life Technologies (Carlsbad, CA, USA). Fetal bovine serum (FBS) was obtained from HyClone (Shanghai, China). The Cell Counting Kit-8 (CCK-8) kit and Calcein AM/Propidium Iodide (PI) were purchased from Beyotime Biotechnology (Shanghai, China). The LIVE/DEAD BacLight Viability Kit was obtained from Thermo Fisher Scientific (Waltham, MA, USA). Phosphate buffer (PBS), 4% paraformaldehyde, and Masson’s Stain solution were obtained from Solarbio (Beijing, China). The Eastep Super Total RNA Extraction Kit was provided by Promega (Shanghai, China), and the Perfect Real-Time RT reagent kit was purchased from Takara Bio (Dalian, China). An enzyme-linked immunosorbent assay (ELISA) Kit for BMP-2 was obtained from Shanghai Haling Biological Technology Co. Ltd. (Shanghai, China); 2,7-dichlorodihydrofluorescein diacetate (DCFH-DA), fluorescent dyes, and fluorescent secondary antibodies were obtained from Sigma-Aldrich (St. Louis, MO, USA). Antibodies were supplied by Abcam (Cambridge, UK).

### 2.2. Hydrogel Preparation and Characterization

TPA was prepared based on a previous report [25]. N, N, N′, N′-tetramethyl-1,3-propanediamine (0.1 g) and 4-(bromomethyl) phenylboronic acid (0.5 g) were dissolved in 10 mL dimethylformamide and then stirred at 60 °C for 16 h. The obtained mixture was added to cold tetrahydrofuran and rinsed three times with tetrahydrofuran. Subsequently, the synthesized TPA was freeze-dried and stored at room temperature after purification to prepare the hydrogel. The hydrogel was immediately prepared by mixing 10 mL PVA (50 mg/mL in PBS) and 10 mL TPA (50 mg/mL in PBS).

To observe the morphology of the hydrogel, scanning electron microscopy (SEM) (JEOL JSM-6700F, Tokyo, Japan) was used at an acceleration voltage of 3 kV. The rheology of the hydrogels was measured using a rheometer (TA Instruments-Waters LLC, New Castle, DE, USA) at room temperature. To investigate the degradation rate in vitro, we immersed the lyophilized hydrogel (200 mg) in PBS and incubated it at 37 °C. To avoid bacterial growth during the degradation period, we added 0.02 wt% sodium azide to the samples. The degradation rate of the hydrogel was analyzed based on the residual amount of hydrogel at the scheduled time points.

### 2.3. H_2_O_2_ Scavenging Capacity of the Hydrogel

The H_2_O_2_ eliminating capacity of the hydrogel was investigated using the typical Ti(SO_4_)_2_ colorimetric method, as previously reported [26]. In simple terms, 1 mL hydrogel and 1 mL H_2_O_2_ (1 mM) were mixed together and cultured in PBS at 37 °C. Subsequently, 24% Ti(SO_4_)_2_ (1.33 mL) and H_2_SO_4_ (8.33 mL) were added into 50 mL deionized water to prepare the Ti(SO_4_)_2_ solution. At pre-set time points, 100 µL of supernatant samples was collected and added to 200 µL Ti(SO_4_)_2_ solution. The concentration of H_2_O_2_ was evaluated by measuring absorbance at 405 nm. In addition, the intracellular H_2_O_2_ scavenging capacity of the hydrogel was monitored using a reactive oxygen species (ROS)-specific probe DCFH-DA. Briefly, resuspended BMSCs were seeded in 24-well plates (5 × 10^4^ cells/well) pre-coated with 200 μL hydrogel and incubated with 100 μM H_2_O_2_. After three days of culture, DCFH-DA (10 μM) was transferred to the cell samples and cultured for 20 min at 20 °C. Finally, the fluorescence intensity, indicating the accumulation of intracellular ROS, was detected and filmed under the fluorescence microscope (IX53, Olympus, Tokyo, Japan).

### 2.4. Drug Loading and Release

To prepare the drug-loaded hydrogel, we dissolved 0.2 mg BMP-2 or 0.5 mg vancomycin in 10 mL PVA (50 mg/mL in PBS) and stirred it thoroughly. The above solution was then mixed with 10 mL TPA (50 mg/mL in PBS) to obtain a drug-loaded hydrogel. To evaluate the release curves of BMP-2 and vancomycin in the hydrogel, we transferred 2 mL hydrogel samples to sterile glass bottles containing 5 mL PBS at 37 °C. At pre-set time intervals, 5 mL PBS was collected and preserved at −20 °C, and another 5 mL fresh PBS was transferred to the glass bottles. The concentration of BMP-2 in the extraction solution was detected by an ELISA kit based on the manufacturer’s instructions, and the mass of released vancomycin was monitored using high-performance liquid chromatography (Agilent 1100, Waldbronn, Germany) at 280 nm. Finally, the cumulative drug release concentrations were analyzed using a standard curve to obtain the release profiles.

### 2.5. Cytocompatibility

To monitor the biocompatibility of the drug-loaded hydrogel, a CCK-8 assay and Calcein-AM/PI staining were performed based on the manufacturer’s protocols. Specifically, BMSCs were seeded in the 24-well plates (at the density of 5 × 10^4^ cells/well) pre-coated with 200 μL hydrogel and BMP-2-and/or vancomycin-loaded hydrogel, abbreviated as Gel, BMP@Gel, Van@Gel, and Dual@Gel, respectively. BMSCs seeded without hydrogel were used as the control group. On the first, fourth, and seventh days after incubation, CCK-8 assays were carried out. Simply put, 10% volume of CCK-8 reagent was transferred into the samples after changing the culture medium. After incubation for 2 h, a 100 μL sample from different groups was collected and transferred to the 96-well plates, and the absorbance was monitored at 450 nm by a Microplate Reader (Thermo Fisher Scientific, MA, USA). On the fourth day after incubation, the cells were cultured with Calcein-AM solution for 15 min and then cultured with PI solution for 5 min at room temperature. The cells were detected visually and imaged using a fluorescence microscope, and the survival rates of the samples were calculated using ImageJ software.

### 2.6. In Vitro Osteogenic Induction of the Drug-Loaded Hydrogel

To detect the capability of the drug-loaded hydrogel to induce osteogenic differentiation, we cultured the attached BMSCs in different groups in the 24-well plates (at the density of 5 × 10^5^ cells/well) in an osteogenic induction medium. After induction for 14 days, calcium nodule deposition in the BMSCs was observed using Alizarin Red staining in line with the manufacturer’s instructions. In addition, 10% cetylpyridinium chloride was transferred to the samples to lysis the deposited calcium nodules for subsequent semi-quantitative evaluation at 562 nm by a microplate reader. ALP activity was investigated using an ALP Assay Kit in line with the manufacturer’s protocols, and the optical density (OD) of the ALP protein was evaluated using a microplate reader at 520 nm.

Furthermore, the levels of osteogenic related genes, including runt-related transcription factor-2 (*Runx-2*), bone sialoprotein (*BSP*), type I collagen (*COL-1*), and osteocalcin (*OCN*), were studied by real-time quantitative PCR (RT-qPCR). The primer sequences of these genes are listed in Table 1. Total RNA was collected by TRIzol reagent, and cDNA was synthesized in reverse transcription reaction by 1 ug total RNA using a Prime Script RT reagent kit. The expression of target genes was detected by qPCR through the use of the SYBR Premix Ex Taq II kit in line with the manufacturer’s protocols. The amplification and performance of RT-qPCR were conducted using 2× Fast SYBR Green Master Mix (Roche Diagnostics, Basel, Switzerland) and analyzed using a LightCycler 480 (Roche Diagnostics). Relative mRNA expressions were normalized by GAPDH and analyzed using the 2^−ΔΔCt^ method.

### 2.7. In Vitro Antimicrobial Evaluation

For antimicrobial research, 1.0 mL of bacterial suspension containing 1.0 × 10^8^ CFUs of *S. aureus* and *MRSA* was inoculated into tubes pre-coated with different hydrogels. The bacteria in tubes were incubated in Luria Bertani (LB) medium at 37 °C and 100 rpm. After incubation for 24 h, the LIVE/DEAD BacLight Bacterial Viability Kit was applied to double-stain the bacteria based on the manufacturer’s instructions. The samples were stained with the dilute dye solution for 15 min in darkness at room temperature and immediately observed and filmed using a fluorescence microscope. The survival rates of the bacterial were calculated using Image J software (V1.8.0.112). Subsequently, 100 μL solution from different samples were collected and the absorbance of bacterial solution was evaluated by a microplate reader at 600 nm.

### 2.8. Preparation of 3D-Printed Implants

Microporous titanium alloy implants were printed layer by layer using an EBM system (Q10, Arcam, Mölnlycke, Sweden) based on previous reports [27,28]. The fundamental parameters of the designed implants were defined as 800 μm for pore size, 70% for porosity, and 300 μm for strut size. The macroscopic shape of the implants was cylindrical, that is, 6 mm in diameter and 10 mm in height. To prepare the 3D-printed implants filled with the drug-loaded hydrogel, we first inserted the porous titanium alloy implants into the TPA solution-containing chamber. Subsequently, BMP-2 or vancomycin-containing PVA solution with equal volumes was added to the chamber. Drug-loaded hydrogel-modified 3D-printed implants were obtained after uniform mixing for approximately 10 min.

### 2.9. Animal Procedures

Forty New Zealand white rabbits (six-month-old females) were used in the in vivo infection model for implant transplantation. General anesthesia was performed by administering 3% (*w*/*v*) pentobarbital at a 50 mg/kg dose. After skin preparation and disinfection, longitudinal incisions were made in the distal femur to expose the lateral condyle layer by layer. Subsequently, a cylindrical bone defect with a diameter of 6 mm and a depth of 10 mm was prepared using a bone drill. After, 500 μL bacterial suspension with 1.0 × 10^6^ CFUs of *S. aureus* and *MRSA* were injected into the bone defects. Then, different hydrogel-modified 3D-printed implants were precisely transferred into the prefabricated bone defects. The surgical incisions were sutured layer-by-layer by the absorbable sutures without postoperative antibiotic administration.

Peripheral blood was drawn through the vein at predetermined time points for white blood cell (WBC) detection and neutrophil analysis to evaluate systemic infection. Six weeks after prosthesis implantation, the animals were executed, and the femur samples were collected after soft tissue removal for sequential detection. The rabbits were intramuscularly injected with 8 mg/kg of calcein on the 14th and fourth day before the execution.

### 2.10. Micro-Computed Tomography (Micro-CT)

For the analysis of bone regeneration and ingrowth, the bone samples were scanned using a micro-CT scanner (SkyScan 1076 scanner, Kontich, Belgium) at 90 kV voltage, 114 mA current, and 18 μm pixel size. The original defect region was set as the region of interest (ROI) for quantitative analysis and 3D reconstruction. To detect the quality of regenerative bone tissue, some parameters, including bone volume/tissue volume ratio (BV/TV), trabecular number (Tb.N), trabecular thickness (Tb.Th), and trabecular separation (Tb.Sp) of the columnar-shaped ROI were measured using micro-CT auxiliary software (NRecon version 1.6.6).

### 2.11. Histological Evaluation

The femoral samples were fixed in 4% paraformaldehyde, embedded in methyl methacrylate without decalcification, and subsequent sectioned into 150–300 μm slices. The slices were ground, polished to 40–50 μm, and observed under a fluorescence microscope to analyze the mineral apposition rate (MAR). The slices were then stained with Masson’s trichrome stain to assess bone ingrowth.

### 2.12. Push-Out Test

The bond strength between the host bone and the implant was investigated via standard push-out detection using an AG-A20 KNA dynamic testing machine (Shimadzu, Kyoto, Japan). Briefly, bone samples were fixed on the experimental platform. The indenter was pushed parallel to the long axis of the implant at a constant speed (0.1 mm/s). The force of the implant detached from the host bone was recorded as the peak push-out force.

### 2.13. Immunofluorescence

After the implants’ push-out, the remaining bone tissues around the implants were preserved and fixed in 4% paraformaldehyde. Bone samples were decalcified in Morse’s solution for five weeks. After complete decalcification, the tissue samples were embedded in paraffin for general histological analyses to produce ~5 μm thickness slices for subsequent immunofluorescence staining. Briefly, the slices were blocked by 3% BSA in PBS containing 0.2% Triton X-100 for 1 h and then cultured with primary antibodies, such as anti-Runx-2 (1:200) plus anti-BSP (1:250), anti-COL-1 (1:200), and anti-OCN (1:150) at 4 °C overnight. After being washed with PBS for 3 times, the slides were incubated with appropriate secondary antibodies for 1 h and DAPI for 5 min at room temperature. Finally, the cell samples observed and filmed by a fluorescence microscope and analyzed through the use of Image J software.

### 2.14. Statistical Analysis

All results were calculated as mean ± standard deviation (SD) and analyzed using Student’s *t*-test between two different groups or one-way analysis of variance (ANOVA) among groups using SPSS (version 19.0; SPSS Inc., Chicago, IL, USA). *p* < 0.05 was considered statistically significant. All experiments were independently repeated at least 3 times.

## 3. Results

### 3.1. Preparation and Characterization of the Hydrogel

**Figure 1:**
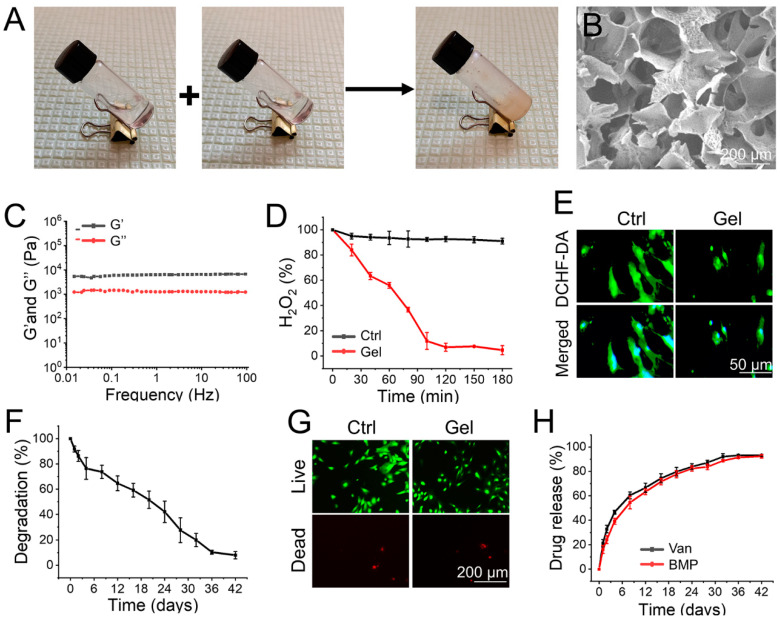
(**A**) The images of the gelation process. (**B**) The SEM observation of hydrogel. (**C**) Frequency spectra of G′ and G″ moduli of the hydrogel. (**D**) Decomposition of H_2_O_2_ (1.0 M) with and without hydrogel. (**E**) The evaluation of ROS scavenging by DCFH-DA staining. (**F**) The in vitro degradation of hydrogel. (**G**) Calcein AM/PI staining of cells. (**H**) Release profiles of vancomycin and BMP-2 from the hydrogel.

As shown in Figure 1A, the supramolecular hydrogel, cross-linked by reactions between phenylboronic acid and alcohol hydroxyl groups, was instantly prepared by mixing TPA and PVA in a one-step process. The synthetic route for the hydrogel was simple and did not involve the production of toxic substances. As shown in Figure 1B, the hydrogel displayed an interconnected network structure under the SEM, and the diameter of micropores was 100 to 200 μm. The interconnected porous structure may benefit oxygen delivery and nutrient transport, thereby inducing cell communication and promoting cell survival, which is beneficial for the biological applications of these materials [29]. The rheological analysis further demonstrated that the hydrogel was successfully prepared (Figure 1C).

The time-dependent H_2_O_2_ scavenging capacity of the hydrogel was subsequently explored. The concentration of H_2_O_2_ remained at its initial value in the control group. However, after incubation with the prepared hydrogel, approximately 45% of H_2_O_2_ in the solution was decomposed within 60 min, and nearly 93% of H_2_O_2_ was eliminated within 120 min (Figure 1D). Furthermore, intracellular ROS in BMSCs was detected using DCFH-DA (a ROS-specific probe) to evaluate the ROS-scavenging ability of the hydrogel. As shown in Figure 1E, BMSCs cultured with the hydrogel indicated decreased fluorescence intensity compared to the control group, demonstrating that the prepared hydrogel could lighten the intracellular ROS accumulation effectively. ROS scavenging hydrogels have potential applications in the microenvironment of bone defects accumulated with ROS. For example, Ding et al. prepared an EGCG-based hydrogel as a protective carrier to regulate stem cell activity and promote bone integration of 3D-printed porous titanium prosthesis by clearing the excessive accumulation of ROS in the bone microenvironment [30]. Furthermore, the degradation rate of the implanted materials is an important factor for their biomedical application. The change in the dry weight of the hydrogel indicated that it was almost completely degraded within 42 days (Figure 1F). The prepared hydrogel also had excellent biocompatibility, which means that the BMSCs cultured with hydrogel maintained cell viability similar to that of the control group (Figure 1G).

The release profiles of vancomycin and BMP-2 from the hydrogel were observed over 42 days. As shown in Figure 1H, in the first 48 h, the percentages of released vancomycin and BMP-2 were 32.8% ± 2.9% and 24.2% ± 3.2%, while the release profiles gradually stabilized in the ensuing days, indicating sustained release profiles of the loaded drugs. In the initial stage, drugs adsorbed on the hydrogel surface by electrostatic interactions had slightly faster release rates. Soon afterward, in the sustained release step, hydrogel degradation became the leading factor in regulating drug release in the carriers. The orderly and stable degradation rate of the hydrogel enabled the continuous release of vancomycin and BMP-2 [31]. It is worth noting that the release rate of vancomycin was slightly faster than that of BMP, which may pave the way for growth factors in the infected environment to play a better role in osteogenesis.

### 3.2. Biocompatibility and Osteogenic Differentiation of BMSCs in Drug-Loaded Hydrogel

**Figure 2:**
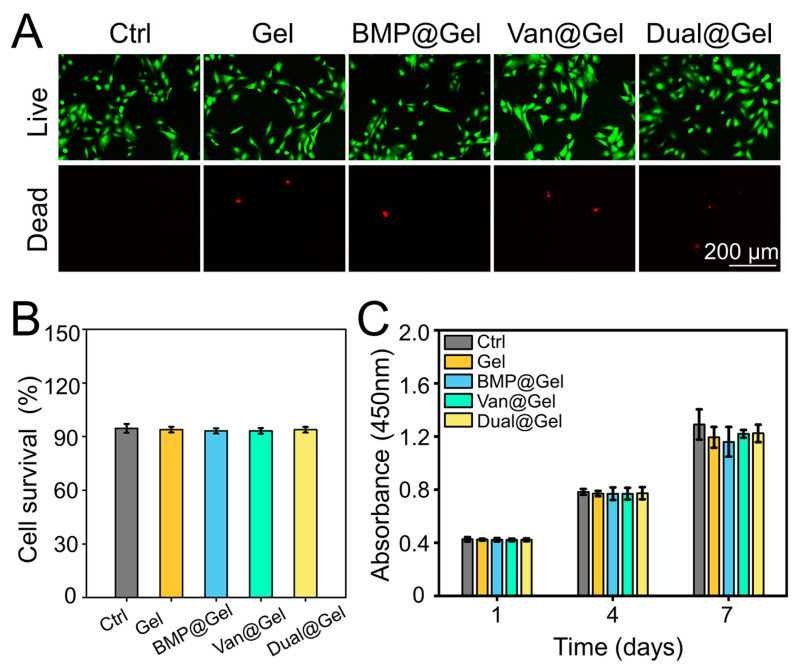
(**A**) Calcein AM/PI staining of cells in different groups. (**B**) Quantitative analysis of survival rates of cells in different groups based on Calcein AM/PI staining. (**C**) Proliferation of BMSCs in the different groups at predetermined time intervals.

The biocompatibility of BMSCs in drug-loaded hydrogels was investigated using Calcein-AM/PI staining detection and a CCK-8 assay. The results indicated that BMSCs in all groups displayed green fluorescence, suggesting that almost all living cells were stained with the Calcein AM dye (Figure 2A). Quantitative analysis showed that the cell survival rates in the control, Gel, BMP@Gel, Van@Gel, and Dual@Gel groups were 94.6% ± 2.4%, 93.9% ± 1.6%, 93.2% ± 1.4%, 93.2% ± 1.5%, and 93.8% ± 1.5%, respectively (Figure 2B). As exhibited in Figure 2C, BMSCs co-cultured in the different groups proliferated gradually without a significant difference at days 1, 4, and 7, showing that the vancomycin or BMP-2-loaded hydrogel had good biocompatibility.

**Figure 3:**
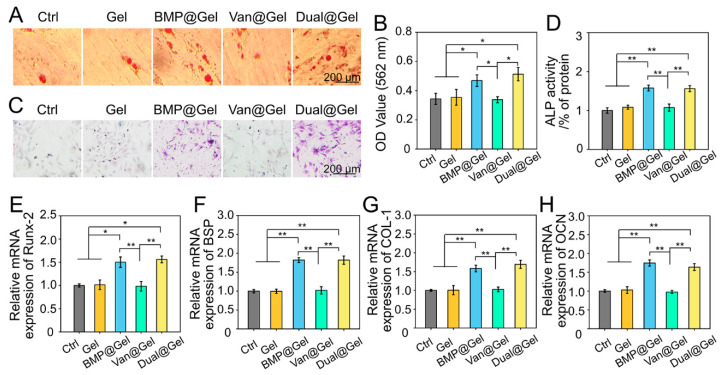
(**A**) Representative photographs of Alizarin Red staining. (**B**) Semi-quantitative analysis of cell mineralization based on Alizarin Red staining. (**C**) Representative images of ALP staining. (**D**) Quantitative analysis of ALP activity of BMSCs. (**E**–**H**) The levels of various osteogenic-related genes expression in BMSCs (* *p* < 0.05, ** *p* < 0.01).

BMSCs have the potential for self-replication and multidirectional differentiation and are found in the mammalian bone marrow matrix [32]. The ability to induce BMSCs recruitment to injured regions and osteogenic differentiation is a vital problem for bone tissue engineering biomaterials [33]. Intracellular calcium nodule deposition is an important feature of osteogenic differentiation, as shown by Alizarin Red staining [30]. Under the influence of BMP-2, there were many calcium nodules in the BMP@Gel and Dual@Gel groups 14 days after osteogenesis induction incubation. However, in the control, Gel, and Van@Gel groups, limited calcium nodule deposition was observed in the samples (Figure 3A). Subsequently, as shown in Figure 3B, semi-quantitative analysis of intracellular mineralization further confirmed that stronger absorbance was detected in the BMP@Gel and Dual@Gel groups than in the control, Gel, and Van@Gel groups (*p* < 0.05). These outcomes demonstrated that the BMP-2-loaded hydrogel increased calcium nodule deposition. The upregulation of ALP activity is an important step in the initial stage of BMSCs osteogenic differentiation [34]. As shown in Figure 3C,D, ALP staining and activity detection indicated that the ALP activity in the BMP@Gel and Dual@Gel groups was significantly higher than that in the control, Gel, and Van@Gel groups (*p* < 0.01).

To test the function of the drug-loaded hydrogel on osteogenic differentiation at the transcription level, critical osteogenic genes, including *Runx-2*, *BSP*, *COL-1*, and *OCN*, were evaluated by RT-qPCR. Activation of *Runx-2* expression is critical in the initial stage of osteogenic differentiation, and *Runx-2* can upregulate the levels of *OCN* and osteopontin (*OPN*) [35]. As another iconic marker of osteogenic differentiation of BMSCs, *COL-1* is an indicator of osteoblast mineralization, indicating bone formation capacity [36]. In addition, *OCN*, which is synthesized by osteoblasts and suggests osteogenic maturation, is an important late osteogenic indicator [37,38]. As shown in Figure 3E,F, *Runx-2*, *BSP*, *COL-1*, and *OCN* expression in the BMP@Gel and Dual@Gel groups were significantly upregulated compared to the control, Gel, and Van@Gel groups.

Owing to the positive influence of BMP-2 on inducing BMSCs osteogenic differentiation and promoting bone regeneration, various BMP-2-loaded bone substitutes have been developed as potential orthopedic implants [39,40,41]. Herein, BMSCs were co-cultured with BMP-2-loaded hydrogel, and the osteogenic differentiation potential of the cells was fully mobilized, demonstrating significantly enhanced calcium nodule deposition, ALP activity, and upregulated osteogenesis-related gene expression.

### 3.3. Antibacterial Assessment

**Figure 4:**
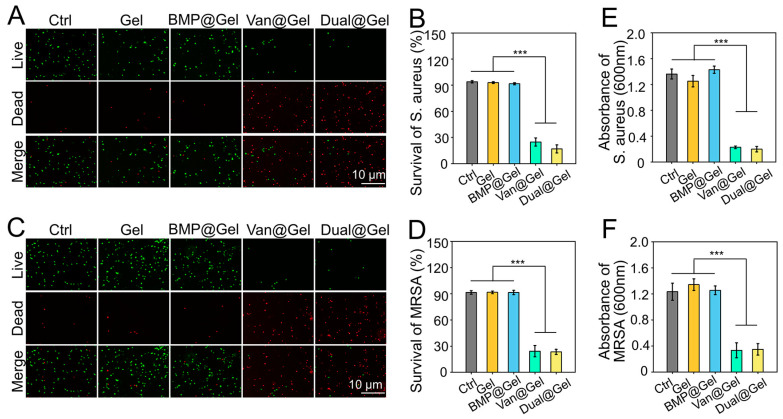
(**A**) BacLight dead/live staining of *S. aureu*s treated with different samples for 24 h. (**B**) Quantitative analysis of survival rates of *S. aureu*s. (**C**) BacLight dead/live staining of *MRSA* treated with different samples for 24 h. (**D**) Quantitative analysis of survival rates of *MRSA*. (**E**) Proliferation of *S. aureus* by optical density measurement. (**F**) Proliferation of *MRSA* by optical density measurement (*** *p* < 0.001).

As common pathogenic microorganisms of bone infection, *S. aureus* and *MRSA* were applied to detect the antibacterial efficacy of drug-loaded hydrogels [42]. Live bacteria have unblemished membranes that can be stained green; on the contrary, dead bacteria with damaged membranes are stained red using the LIVE/DEAD BacLight Viability Kit [43]. After incubation for 24 h, the staining results indicated that multitudinous *S. aureus* cultured in the control, Gel, and BMP@Gel groups showed close to green fluorescence, indicating that these materials possessed negligible antibacterial capacity. However, bacteria incubated in the Van@Gel and Dual@Gel groups emitted close to red fluorescence (Figure 4A), indicating that the antimicrobial effect of the vancomycin-loaded hydrogel was significant. As indicated in Figure 4B, quantitative analysis of survival rates of *S. aureu*s in the control, Gel, BMP@Gel, Van@Gel, and Dual@Gel groups were 93.9% ± 1.3%, 92.9% ± 1.1%, 91.7% ± 1.1%, 24.8% ± 4.6%, and 16.8% ± 4.5%, respectively. In addition, the vancomycin-loaded hydrogel showed an excellent antibacterial effect on *MRSA*, another common bacterium for peri-implant infection (Figure 4C). The analysis of survival rates of *MRSA* in the control, Gel, BMP@Gel, Van@Gel, and Dual@Gel groups were 91.5% ± 2.0%, 91.7% ± 1.5%, 91.5% ± 2.5%, 24.3% ± 6.5%, and 23.4% ± 2.9%, respectively (Figure 4D). In addition, bacteria presence in the medium were tested by optical density measurement, and the results indicated that Van@Gel, and Dual@Gel groups significantly inhibited the bacterial proliferation (Figure 4E,F).

### 3.4. In Vivo Anti-Infection Effects of the Drug-Loaded Hydrogel

**Figure 5:**
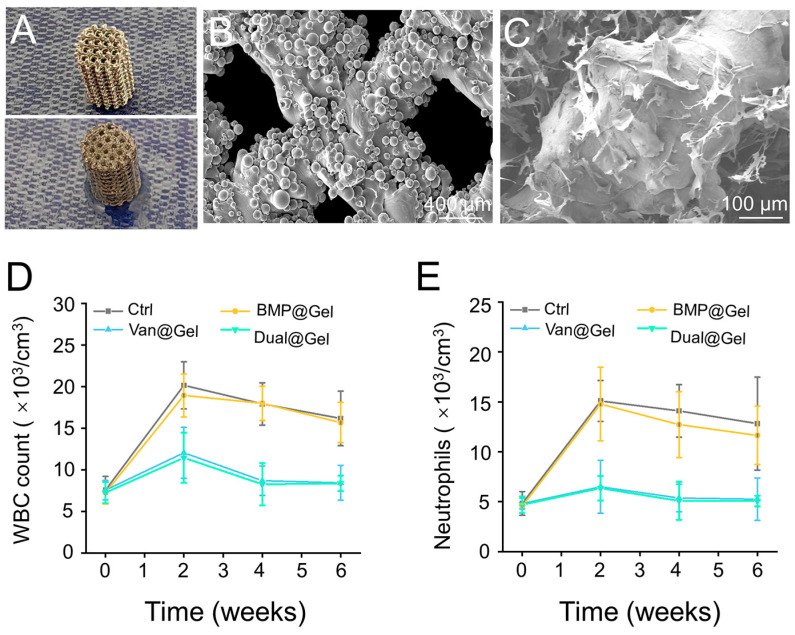
(**A**) The visual photos of titanium alloy implant and hydrogel modified implant. (**B**) The SEM image of the microporous titanium alloy implant. (**C**) The SEM image of hydrogel modified implant. (**D**) WBC count in the peripheral blood to monitor systemic infection. (**E**) Neutrophil analysis in the peripheral blood to monitor systemic infection.

The visual photos of prepared titanium alloy implant and hydrogel modified implant were displayed in Figure 5A. The SEM images of the microporous titanium alloy implant revealed that the pore size of the scaffold was ~800 μm (Figure 5B). In addition, after modification, the hydrogel fills all the empty space in porous structure of the implant (Figure 5C). The management of peri-implant infections remains a formidable clinical challenge. A series of therapeutic strategies have been applied, including antibiotic spacers, surgical debridement, and vascularized bone grafts. [44,45]. However, due to the lack of biodegradability and osteoinductivity, conventional bone cement-loaded antimicrobial agents have not been prioritized in managing infected bone defects [46]. Therefore, bone substitutes with excellent bone conductivity and significant antibiotic activity can be used as implants for contaminated bone defects [47]. However, owing to the multiple drug resistance of bacteria and insufficient osteoinduction, there are still no effective bone substitutes in the market [48]. A hydrogel with dual antibacterial and osteogenic induction properties was designed to address these challenges in managing infected bone defects. As shown in Figure 5D, the severity of systemic infection was assessed using the peripheral blood WBC count and neutrophil detection. These values were within the physiological range at 0 weeks, that is, before bacterial implantation. However, after implantation, the WBC counts in the control and BMP@Gel groups increased continuously and were higher than those in the Van@Gel and Dual@Gel groups. This phenomenon indicated that the local release of vancomycin inhibited bacterial proliferation and spread. Another indicator of infection status, neutrophil count, showed similar results (Figure 5E).

*S. aureus* is one of the most common bacteria in cases of bone infection [49]. It has been demonstrated that *S. aureus* is found in 80%–90% of patients with suppurative osteomyelitis [50]. The proportion of *MRSA* in bone infections is also increasing [51,52]. Vancomycin is a glycopeptide antibiotic with a strong antibacterial effect on gram-positive bacteria, especially against infectious diseases caused by drug-resistant bacteria. The antibacterial mechanism of vancomycin mainly involves the inhibition of bacterial cell wall synthesis. It can also change the permeability of bacterial cell membranes and affect RNA synthesis in pathogenic bacteria [53,54]. Herein, a study of infected bone defects in rabbits demonstrated that vancomycin-loaded hydrogel could effectively restrain the local proliferation of bacteria and systemic infection.

### 3.5. Bone Regeneration and Integration in Contaminated Bone Defect

**Figure 6:**
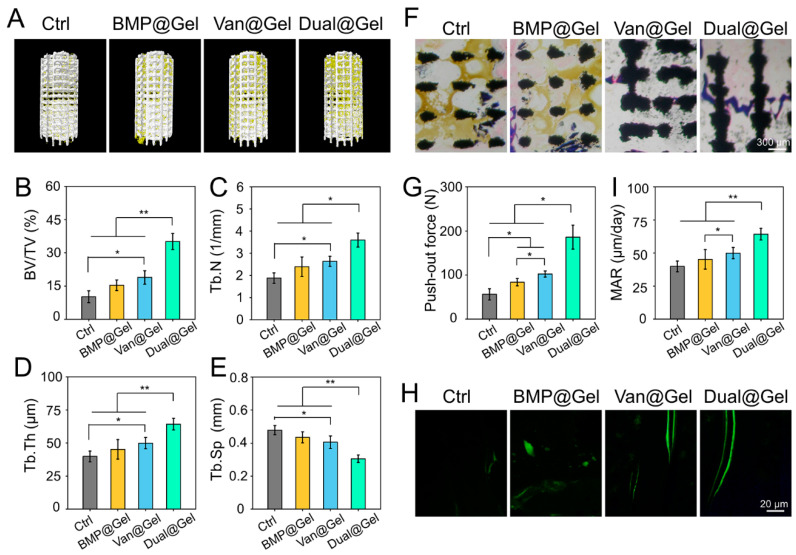
(**A**) Representative 3D reconstruction photographs of bone regeneration around the implant. (**B**–**E**) Analysis of bone morphological parameters, including BV/TV, Tb.N, Tb.Th, and Tb.Sp around the implant based on Micro-CT scanning. (**F**) Representative Masson staining images in the region of bone defects. (**G**) Evaluation of bone integration by pull-out biomechanical testing. (**H**) Representative images of calcein fluorescence double-labeling. (**I**) MAR analysis based on calcein double-labeling (* *p* < 0.05 and ** *p* < 0.01).

Local infection can severely hinder the bone integration process between the host bone and the implant. As an ideal bone substitute for managing infected bone defects, promoting bone healing is vital in addition to controlling the infection [55,56]. Therefore, micro-CT scanning and 3D reconstruction were conducted 6 weeks after implant transplantation to detect the therapeutic effects of the drug-loaded hydrogel on bone integration. As shown in Figure 6A, the representative images showed that the porous implant simultaneously loaded with vancomycin and BMP-2 (Dual@Gel group) suggested better integration between the host bone and implant, as demonstrated by obvious bone regeneration around the prosthesis interface compared to the other groups. Although BMP@Gel had an enhanced osteogenesis effect with BMP-2, it lacked anti-infection factors, and bone regeneration was unsatisfactory and limited by local and systemic infection status. Data obtained from micro-CT scanning were analyzed quantitatively to reveal bone regeneration around the implant further. The BV/TV values of the control, BMP@Gel, Van@Gel, and Dual@Gel groups were 10.1 ± 2.7%, 15.3 ± 2.4%, 18.8 ± 3.0%, and 35.1 ± 3.6%, respectively (Figure 6B). In addition, other quantitative analysis parameters indicated similar results, showing that the Dual@Gel group showed the highest values of Tb.N and Tb.Th and the lowest values of Tb.Sp among all the groups (Figure 6C–E).

Sufficient bone regeneration and integration between the host bone and the implant can decrease postoperative complications after implant transplantation, especially catastrophic revision surgery. Undecalcified tissue sections were prepared and stained with Masson’s dye for histological evaluation to investigate bone ingrowth into the pores and integration of implants. According to the representative images in Figure 6F, the regenerated bone tissues could be observed around the interface on the porous implant, even growing into interconnected pores in the Dual@Gel group. However, because of the lack of antibacterial ability of vancomycin, simple loading of BMP-2 could not achieve satisfactory results in bone regeneration in infected bone defects, probably due to the inactivation of released BMP-2 in the infected environment and the excessive inflammatory state preventing bone repair. Specifically, in the control and BMP@Gel groups, yellow pus accumulated in the pores, which was a sign of infection around the implant. Biomechanical push-out tests were performed to further detect bone integration between the implant and host bone tissue. The values of push-out peak force in the control, BMP@Gel, Van@Gel, and Dual@Gel groups were 56.1 ± 12.7 N, 83.6 ± 8.3 N, 102.3 ± 6.8 N, and 185.9 ± 27.1 N, respectively (Figure 6G). These outcomes indicated that the 3D-printed implants filled with vancomycin and BMP-2-loaded hydrogel achieved more bone regeneration and powerful bone integration in the infected bone defects.

**Figure 7:**
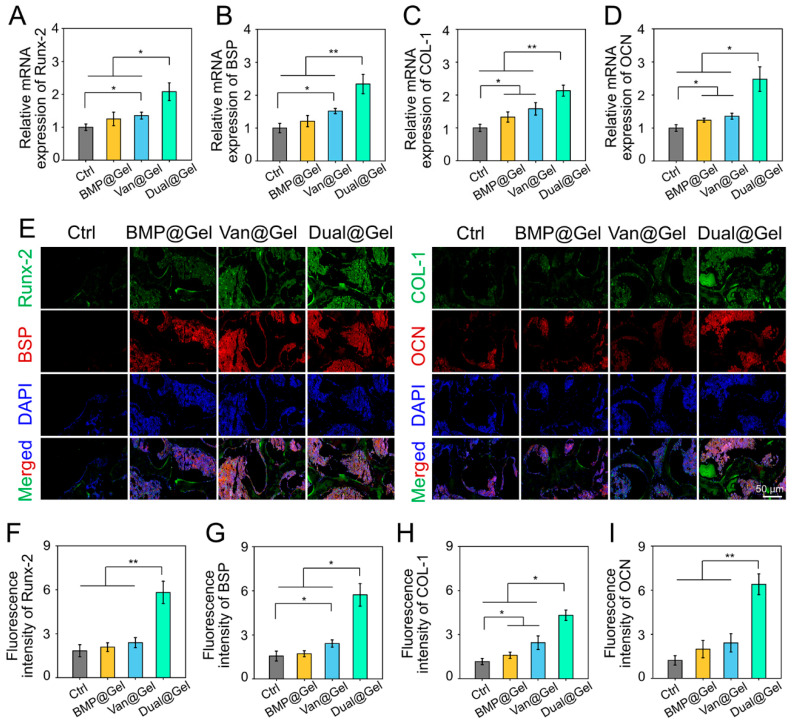
(**A**–**D**) The mRNA expression levels of various osteogenic-related genes in the regenerated bone tissues. (**E**) Immunofluorescence staining of bone tissues around the implant. (**F**–**I**) Quantitative analysis of various osteogenic-related proteins based on the immunofluorescence (* *p* < 0.05, ** *p* < 0.01).

As exhibited in Figure 6H, double-labeling of calcein fluorescence was performed to detect the MAR of the bone tissue around the porous implant. The MAR value in the Dual@Gel group was 1.6 μm/day, which was faster than that in the control (0.9 μm/day), BMP@Gel (1.0 μm/day), and Van@Gel (1.2 μm/day) groups 6 weeks after transplantation (*p* < 0.01), suggesting a higher bone formation rate (Figure 6I). In addition, RT-qPCR and immunofluorescence staining of various osteogenesis-related indicators were conducted to detect the cause of drug-loaded hydrogel-enhancing peri-implant osseointegration. RT-qPCR analysis demonstrated that osteogenic genes, such as *Runx-2*, *BSP*, *COL-1*, and *OCN,* in the Dual@Gel group were profoundly enhanced compared with those in the control, BMP@Gel, and Van@Gel groups (Figure 7A–D). Additionally, the content of Runx-2, BSP, COL-1, and OCN in the bone tissues was evaluated by immunofluorescence and displayed in Figure 7E. Among the four groups, the fluorescence intensity of these proteins in the Dual@Gel group had the highest values in bone tissue around the implant (Figure 7F–I). The 3D-printed porous implant modified with the drug-loaded (vancomycin and BMP-2) hydrogel can effectively inhibit bacterial infection around the implant, paving the way for the released BMP-2 in the infected microenvironment to play a role in bone regeneration and integration by upregulating various osteogenic markers.

## 4. Conclusions

In conclusion, we prepared a functional hydrogel assembly using TPA and PVA loaded with antibacterial and osteogenic agents (vancomycin and BMP-2) as a modified material to fill the porous titanium alloy implant. This hydrogel has a significant antibacterial effect on *S. aureus* and *MRSA* and promotes the osteogenic differentiation of BMSCs. Furthermore, the drug-loaded hydrogel can significantly promote bone regeneration and the integration of 3D-printed porous titanium alloy implants in contaminated bone defects. Consequently, drug-loaded, hydrogel-modified porous implants with dual function may have potential applications and clinical transformation significance in the treatment of infected bone defects.

## Ethical Statement

The animal study was reviewed and approved by the Institutional Animal Care and Use Committee (IACUC) of the Shanghai Ninth People’s Hospital, Shanghai Jiao Tong University School of Medicine.

## Figures and Tables

**Table 1:** Primers of target genes.

Target Genes	Primers
*Runx-2*	F: 5′-AGAGTCAGATTACAGATCCCAGG-3′
	R: 5′-TGGCTCTTCTTACTGAGAGAGG-3′
*COL-I*	F:5′-GATGTTGAACTTGTTGTTGCTGAGGG-3′
	R:5′-GGCAGGCGAGATGGCTTATT-3′
*BSP*	F:5′-AAAAGTGAAGGAAAGCGACGAG-3′
	R:5′-CGTGGAGTTGGTGCTGGTG-3′
*OCN*	F:5′-GAACAGACAAGTCCCACACAGC-3′
	R:5′-TCAGCAGAGTGAGCAGAAAGAT-3′
*GAPDH*	F:5′-CTCGTCCCGTAGACAAAATGGT-3′
	R:5′-GAGGTCAATGAAGGGGTCGTT-3′

## Data Availability

The data that support the findings of this study are accessible from the corresponding author upon reasonable request.
